# Ulna hook plate osteosynthesis for ulna head fracture associated with distal radius fracture

**DOI:** 10.1186/s10195-022-00658-3

**Published:** 2022-08-16

**Authors:** Morgan Gauthier, Jean-Yves Beaulieu, Lucille Nichols, Didier Hannouche

**Affiliations:** Division of Orthopaedics and Traumatology, University Hospitals of Geneva, Geneva, Switzerland

**Keywords:** Distal ulna fracture, Plate osteosynthesis, Ulna hook plate

## Abstract

**Background:**

Distal ulna head or neck fracture is commonly associated with distal radius fracture. Treatment of these fractures remains controversial. Plate osteosynthesis is commonly performed. The purpose of this study was to observe clinical and radiological outcomes in ulna hook plate osteosynthesis for distal ulna fracture associated with distal radius fracture.

**Materials and methods:**

This retrospective study between 2010 and 2018 included patients presenting combined displaced distal ulna fracture and distal radius fracture who were treated with ulna hook plate osteosynthesis. Patient evaluation included pain measurement with the visual analog scale, wrist range of motion, grip and pinch strengths, Quick Disabilities of the Arm, Shoulder and Hand (Q-DASH) score, and Mayo wrist score. Preoperative radiographs were reviewed to classify the distal ulna fracture according to Biyani. Bone union was evaluated on postoperative X-rays. At final follow-up, the usual radiographic parameters were measured and distal radioulnar joint (DRUJ) osteoarthritis was assessed.

**Results:**

A total of 48 patients were included. Mean age was 63 years old and mean follow-up was 28 months. According to the Biyani classification, there were 12 type I, 4 type II, 8 type III, and 24 type IV distal ulna fractures. Wrist flexion was 60°, extension 57°, pronation 85°, and supination 80°. Grip strength was 21 kg (86% of the uninjured opposite side). Pinch strength was 6.6 kg (92% of the uninjured opposite side). Clinical scores were very good to excellent, with a mean Q-DASH of 12 and a Mayo wrist score of 90. Discomfort or pain due to the implant that required implant removal was reported in 29%, and was higher in younger patients. Nonunion was observed in two cases and secondary implant displacement in one case. These three cases required secondary intervention with ulna head resection, which was higher in Biyani type IV. DRUJ osteoarthritis was observed in 12 patients (31%) and was higher in older patients.

**Conclusions:**

Ulna hook plate fixation gives good clinical results and a high rate of fracture union, but complications are common. Implant irritation is a frequent complication, especially in young patients, and often requires implant removal.

**Level of evidence: IV:**

## Introduction

Fracture of the distal head or neck of the ulna is an uncommon injury [1–3]. These fractures are rarely isolated, and are frequently associated with distal radius fractures. Distal ulna fractures are combined with distal radius fracture in 5.6% of cases [3]. Their treatment remains controversial. In an unstable or displaced ulna fracture, the distal radioulnar joint is frequently involved, which can lead to instability, a decreased range of wrist motion, and an increased nonunion rate [4–6]. Therefore, several studies have recommended operative treatment for unstable or displaced distal ulna fractures after reduction and fixation of the distal radius fracture [1].

Open reduction and internal fixation with plate osteosynthesis is a common operative treatment [1, 5–9]. Several plates have been used, some of them having a low profile and angular stability, enhancing early mobilization [1, 7]. However, these implants may cause discomfort or pain [1].

Ulna head resection (Darrach’s procedure) is another operative treatment option, especially in older patients with low functional demand or with osteoporosis [1, 10–14]. Kirschner pinning is a less common procedure, as it is unsuitable for stabilizing comminuted fractures and has a high rate of complications, such as Kirschner migration, loss of reduction, discomfort, and infection [7].

The purpose of this study is to report the clinical and radiological outcomes after ulna hook plate osteosynthesis for distal ulna fracture.

## Materials and methods

Inclusion criteria comprised a displaced ulna head fracture associated with a distal radius fracture. Exclusion criteria were an undisplaced ulna head fracture, an isolated ulna styloid fracture, and an isolated distal ulna fracture. From October 2010 to March 2018, 82 consecutive patients with combined distal ulna and distal radius fractures were treated operatively with radius osteosynthesis combined with ulna plate osteosynthesis. Of those 82 patients, 13 had died, 14 were unreachable, and 7 had less than 6 months’ follow-up. A total of 48 patients (59%) were included in this study.

### Surgical technique

The first surgical step consisted of distal radius exposure by a modified Henry approach between the flexor carpi radialis and the radial artery, followed by fracture reduction and stabilization using a volar plate. In the second step, the distal ulna was exposed between the extensor carpi ulnaris and flexor carpi ulnaris, followed by fracture reduction and stabilization using a 2.0-mm locking compression plate (LCP) distal ulna hook plate (Depuy Synthes, West Chester, PA). This plate was placed on the lateral side of the ulna shaft and the pointed hooks of the plate were placed around the tip of the ulna styloid. Postoperative management included an antebrachial cast for 6 weeks and progressive active motion at 6 weeks.

### Postoperative complications

Complications were reported, including discomfort or pain due to the implant, injury of the dorsal cutaneous branch of the ulnar nerve, complex regional pain syndrome (CRPS), and infection. Fracture nonunion or secondary displacements were also reported.

### Clinical evaluation (at last follow-up)

Wrist range of motion was measured with a goniometer. Grip strength and pinch strength were evaluated with hydraulic dynamometers (JAMAR®, Warrenville, IL). Pain was evaluated using the visual analog scale (VAS) [15] at rest and during activity. The patient-related general outcome was measured using the Quick Disabilities of the Arm, Shoulder and Hand (Q-DASH) score [16] and the Mayo wrist score. The best Q-DASH score is 0% and the best Mayo wrist score is 100%.

### Radiographic evaluation (preoperative and at last follow-up)

Preoperative radiographs were reviewed to classify distal ulna fractures according to Biyani [17] and distal radius fractures according to the AO/OTA [18]. Biyani type I is a simple ulna head fracture, type II is an inverted T-fracture, type III is a combined ulna head and ulna styloid fracture, and type IV is a comminuted ulna head fracture. AO/OTA type A is an extraarticular fracture, type B is a partial articular fracture, and type C is a complete articular fracture. Bone union was evaluated on postoperative X-rays. At final follow-up, radial height, ulnar variance, radial inclination, and volar tilt were calculated. Distal radioulnar joint (DRUJ) osteoarthritis was assessed. Ulna plate placement was assessed to confirm its position on the lateral side of the ulna.

### Comparison between younger and older patients

Patients were separated into two groups: patients younger than 65 years (group 1) and patients 65 years old or older (group 2). The following variables were compared between these two groups: mechanism, AO/OTA classification, Biyani classification, range of motion, strength, Q-DASH, complications, and DRUJ osteoarthritis.

### Statistical analysis

The significance of differences between the two groups was assessed using the Mann–Whitney U test. The statistical analysis was performed using StatPlus version 7.3.1 (Addinsoft, NY, USA). The chosen level of evidence was *p* < 0.05.

## Results

Forty-eight patients with a combined displaced distal ulna fracture and radius fracture treated operatively were included. There were 40 females and 8 males with a mean age of 63 years (range 22 to 93 years). Mean follow-up was 28 months (range 6 to 102 months). Mechanism of injury was a low-energy fall in 36 patients and high-energy trauma in 12 patients. There were 41 closed fractures and 7 open fractures (6 cases of Gustilo type I and one case of Gustilo type II). Demographic data are summarized in Table 1﻿.

**Table 1:** Demographic data, postoperative complications, and clinical results at last follow-up

	Patients (*n* = 48)
Sex	40 females, 8 males
Age	63 years (± 18 years)
Weight	68 kg (± 15 kg)
Height	166 cm (± 9 cm)
BMI	25 (± 5)
ASA score	12 ASA-1
	31 ASA-2
	5 ASA-3
Mechanism of injury	
High-energy trauma	12 patients (25%)
Low-energy trauma	36 patients (75%)
Type of fracture	
Closed	41 patients (85%)
Open	7 patients (15%)
Postoperative complications	
Discomfort or pain due to ulna plate requiring implant removal	14 patients (29%)
Hypoesthesia (dorsal branch)	4 patients (8%)
CRPS	1 patient (2%)
Nonunion	2 patient (4%)
Implant displacement	1 patient (2%)
Follow-up	28 months (± 28 months)
Clinical scores	
Q-DASH score	12 (± 18)
Mayo wrist score	90 (± 10)
Visual analog scale (VAS)	
At rest	0.3 (± 1.1)
During activity	0.6 (± 1.3)
Range of motion	
Wrist flexion	60° (± 14°)
Wrist extension	57° (± 14°)
Pronation	85° (± 7°)
Supination	80° (± 12°)
Strength	
Grip strength	21 kg (± 12 kg)
Pinch strength	6 kg (± 3 kg)

*BMI* body mass index, *ASA* American Society of Anesthesiologists, *VAS* visual analog scale, *Q-DASH*: Quick Disabilities of the Arm, Shoulder, and Hand, *CRPS* complex regional pain syndrome

### Postoperative complications

**Fig. 1:**
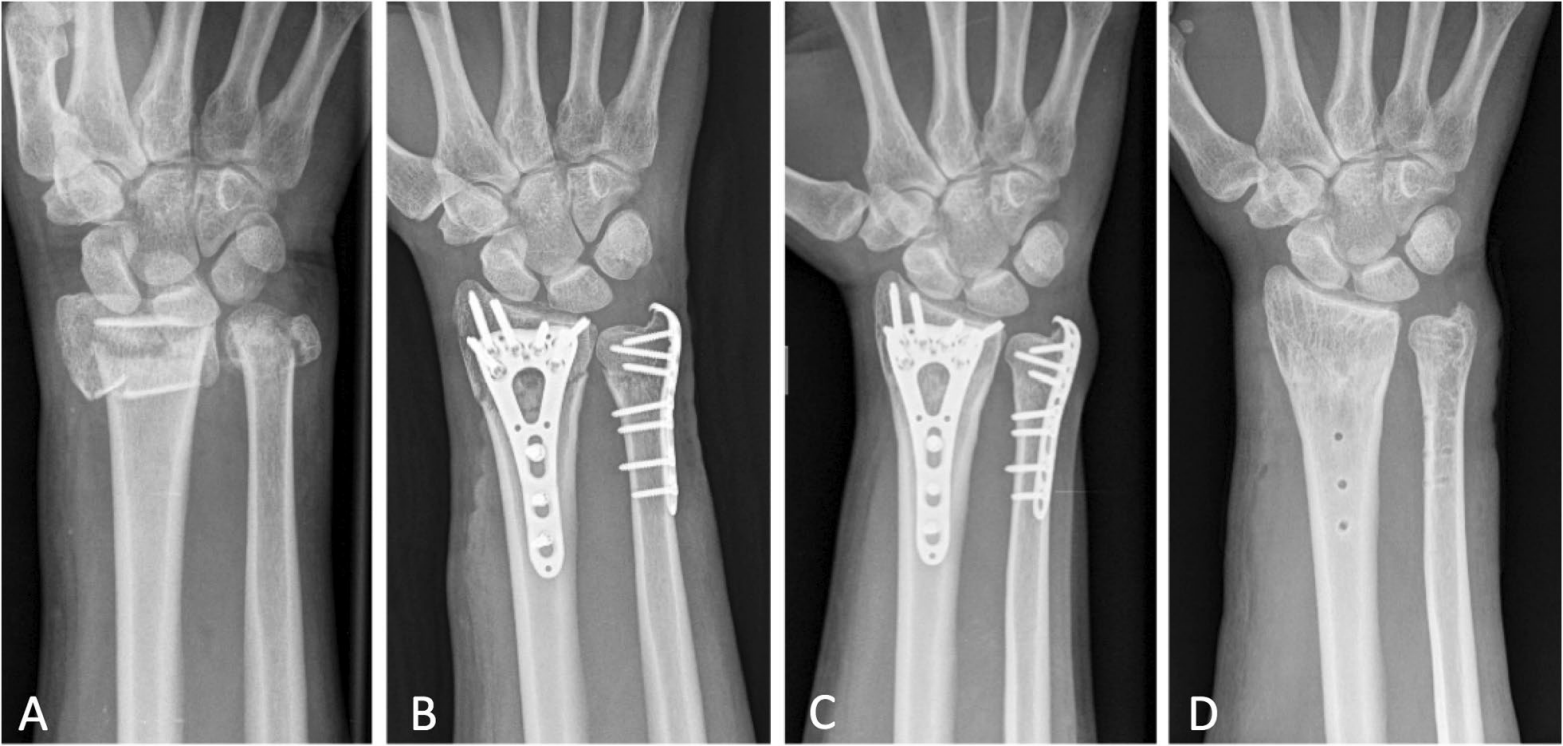
Discomfort after ulna plate osteosynthesis in a young patient. **A** Preoperative radiograph of a 29-year-old female presenting a Biyani type IV right distal ulna fracture combined with a type C distal radius fracture. **B** Immediate postoperative radiograph showing right wrist osteosynthesis with ulna hook plate and radius volar plate. **C** One-year postoperative radiograph showing ulna and radius fracture union. **D** Radiograph after the removal of implants due to ulna plate discomfort

**Fig. 2:**
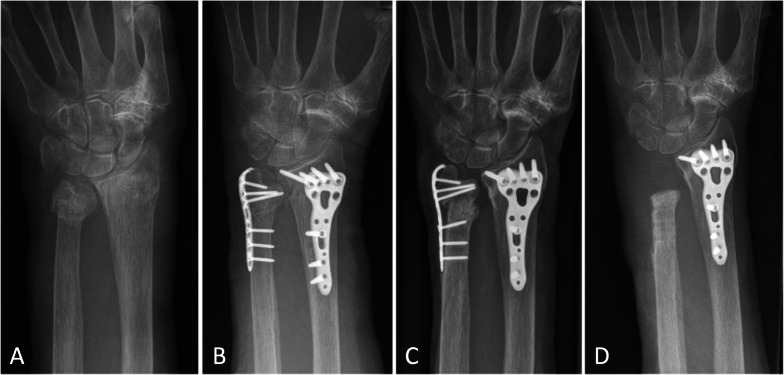
Nonunion after ulna plate osteosynthesis in an old patient. **A** Preoperative radiograph of a 93-year-old female presenting a Biyani type IV left distal ulna fracture combined with a type C distal radius fracture. **B** Immediate postoperative radiograph showing left wrist osteosynthesis with ulna hook plate and radius volar plate. **C** One-year postoperative radiograph showing ulna and radius fracture nonunion. **D** Radiograph after ulna head resection (Darrach) due to ulna nonunion

Discomfort or pain due to implant that required implant removal was reported in 14 patients (29%) (Fig. 1). There was a correlation with age, with younger patients having a higher rate of discomfort. However, no correlation was found with sex, BMI, ASA score, radius fracture type (AO/OTA), or ulna fracture type (Biyani). Hypoesthesia of dorsal cutaneous branch of the ulnar nerve was observed in 4 patients (8%)—permanently in 3 patients and temporarily in 1 patient. One patient developed CRPS. No infection was reported. Nonunion was observed in two cases (Fig. 2) and secondary implant displacement in one case. These three cases required secondary intervention with ulna head resection. Age and ASA score were not correlated with major complications. However, major complications were observed only in Biyani type IV.

### Clinical results (at last follow-up)

At the final follow-up, wrist flexion was 60° (± 14°) and extension was 57° (± 14°). Forearm pronation was 85° (± 7°) and supination was 80° (± 12°). Grip strength was 21 kg (± 12 kg), which was 86% of the uninjured opposite side. Pinch strength was 6.6 kg (± 3 kg), which was 92% of the uninjured opposite side. Clinical results are summarized in Table 1.

Mean VAS at rest was 0.3 (± 1.1) and the VAS during activity was 0.6 (± 1.3). Q-DASH was very good to excellent, with a mean score of 12 (± 18), and the Mayo wrist score was 90 (± 10).

### Radiographic results (preoperative and at last follow-up)

On preoperative X-ray, according to the Biyani classification for distal ulna fractures, there were 12 type I, 4 type II, 8 type III, and 24 type IV fractures. According to the AO/OTA classification for distal radius fractures, there were 21 type A and 27 type C fractures.

At the last follow-up, X-ray analysis showed satisfactory reduction. Radial height was 10.6 mm (± 2.8 mm), ulnar variance was −1.6 mm (± 2.0 mm), radial inclination was 20.6° (± 5.2°), and volar tilt was 4.8° (± 8.7°).

DRUJ osteoarthritis was observed in 12 patients (31%) after the exclusion of 3 patients with ulna head resection, 2 patients with crystal arthropathy, and 4 patients with pancarpal arthritis. DRUJ osteoarthritis was not correlated with Biyani type, but with age (older patients having a higher incidence).

All patients had correct ulna hook plate placement on the lateral side of the ulna.

Radiographic results are summarized in Table 2.

**Table 2:** Radiographic results (preoperative and at last follow-up)

	Patients* n *= 48
Preoperative biyani classification (distal ulna fracture)	
Type I	12 cases (25%)
Type II	4 cases (8%)
Type III	8 cases (17%)
Type IV	24 cases (50%)
Preoperative AO/OTA classification (distal radius fracture)	
A	21 cases (44%)
B	0 cases (0%)
C	27 cases (56%)
Radiographic parameters evaluation at last follow-up	
Radial height	10.6 mm (± 2.8 mm)
Ulnar variance	−1.6 mm (± 2.0 mm)
Radial inclination	20.6° (± 5.2°)
Volar tilt	4.8° (± 8.7°)

### Comparison between younger and older patients

There were 26 patients in group 1 (younger than 65 years) and 22 patients in group 2 (65 years or older) (Table 3). High-energy trauma was found only in younger patients. Fracture type according to Biyani or AO/OTA was not correlated to age. A better range of motion was observed in the younger group. Grip strength and pinch strength were higher in the younger group. Functional outcomes measured with Q-DASH and the Mayo wrist score were similar between groups. There was a higher rate of discomfort or pain due to the implant, which required the removal of the implant, in the younger group. Nonunion was observed in the older group (*n * = 1) and in the younger group (*n* = 1). Implant displacement was only observed in the older group (*n* = 1). The rate of DRUJ osteoarthritis was slightly higher in the older group (45%) than in the younger group (30%).

**Table 3:** Comparison between younger and older patients treated with distal ulna hook plate osteosynthesis

	Group 1 (*n* = 26)	Group 2 (*n* = 22)	
Age	< 65 years	> 65 years	
Mechanism			
High-energy trauma	12	0	
Low-energy fall	14	22	
AO/OTA classification (distal radius fracture)			
Type A	11	10	
Type B	0	0	
Type C	15	12	
Biyani classification (distal ulna fracture)			
Type I	6	6	
Type II	3	1	
Type III	4	4	
Type IV	13	11	
Range of motion			
Wrist flexion	64°	55°	***
Wrist extension	62°	50°	***
Pronation	85°	83°	
Supination	81°	79°	
Strength			
Grip strength	27.2	13.5	***
Pinch strencth	8.2	4.5	***
Q-DASH score	8 (± 13)	17 (± 23)	
Mayo wrist score	90 (± 11)	90 (± 8)	
Complications			
Discomfort or pain due to implant	12 cases	2 cases	***
Hypoesthesia (dorsal branch)	3 cases	1 cases	
CRPS	1 case	0 case	
Nonunion	1 case	1 case	
Implant displacement	0 case	1 case	

*Q-DASH* Quick Disabilities of the Arm, Shoulder and Hand, *CRPS* complex regional pain syndrome; *** stastistical significant difference (p<0.05)

^***^stastistical significant difference (*p* < 0.05)

## Discussion

The ulna hook plate is a common method of stabilizing distal ulna fractures. Several plates have been analyzed in the literature. Good results were obtained using a condylar blade plate [6], while satisfactory outcomes were achieved using a 2.0 mm locking plate [7]. The more recent 2.0 mm LCP ulna hook plate allows a lower plate profile and can also facilitate the reduction of an associated ulna styloid fracture (Biyani type 3 or 4). This ulna hook plate was found to be an effective means of fixation [1, 9, 12].

Our study showed good to excellent outcomes with an ulna hook plate in the case of a distal ulna head fracture. Q-DASH functional scores and Mayo wrist scores were very good to excellent. Satisfactory wrist motion and strength were measured and were comparable to prior studies.

Most authors suggest a conservative treatment in the case of an undisplaced or stable distal ulna fracture. Only one study [19] showed that similar results were achieved with osteosynthesis and conservative treatment of an unstable ulna fracture in patients older than 65 years old [19]. No study has described conservative treatment for younger patients.

Some authors have suggested ulna head resection in cases of comminuted fracture which cannot be fixed with osteosynthesis and associated osteoporosis in low-demand patients of advanced age or patients with dementia. A recent study by Boretto et al. [10] showed that similar results were achieved with osteosynthesis and ulna head resection in patients older than 70 years old. However, they showed a high rate of complications with osteosynthesis.

Major complications such as nonunion or displacement of the implant were observed in three patients. This correlated with the Biyani classification, but not with age or ASA score. This rate is low and comparable to reported results in the literature. Minor complications such hypoesthesia were observed in 6%, and implant irritation was observed in 28%. Implant irritation is not frequently reported in the literature, but one study found that implant irritation occurred in 16% of cases [1]. In our study, younger patients showed implant-related pain more often than older patients. This younger population is typically more active and frequently engage in highly demanding activities and sports. No correlation was found with sex, BMI, ASA score, or fracture type. Moreover, implant discomfort was not due to plate placement because the plate was placed correctly on the lateral side of the ulna in all patients, allowing firm fixation with the hook on the ulna styloid. However, the plate was placed close to the wrist joint, which could explain the high incidence of discomfort or pain in this region in high-demand patients.

DRUJ osteoarthritis was observed in 31% of cases. No study mentioned their rate of osteoarthritis. The DRUJ osteoarthritis diagnosis was made based on the last radiograph. This high rate is probably related to the semiquantitative evaluation (with or without osteoarthritis), and a more precise classification would help with the comparison. Older patients showed a higher rate of DRUJ osteoarthritis than younger patients. This could be explained by trauma differences but also by the increasing rate of osteoarthritis of the wrist with aging.

## Conclusion

In the present study, ulna hook plate fixation yielded good clinical results and a high rate of fracture union, although complications were common. The authors suggest plate osteosynthesis in young and active patients, which allows for strong fixation and a quick return to daily activities. However, patients should be advised that implant irritation is common and often requires implant removal. For older and less active patients, treatment remains controversial. Plate osteosynthesis gives good results with a low incidence of implant irritation, but some major complications can appear (fracture nonunion, secondary implant displacement). Ulna head resection in elderly patients remains a good alternative treatment.

## Data Availability

The datasets used and analyzed during the current study are available from the corresponding author on reasonable request.

## Abbreviations

LCP: Locking compression plate
VAS: Visual analog scale
Q-DASH: Quick disabilities of the arm, shoulder and hand (Q-DASH)
CRPS: Complex regional pain syndrome
DRUJ: Distal radioulnar joint
ASA: American Society of Anesthesiologists
